# Biopsy-Proven Glomerulopathies in Romania: A 10-Year Nationwide Study

**DOI:** 10.3390/life15060938

**Published:** 2025-06-11

**Authors:** Andreea Covic, Mihai Onofriescu, Flaviu R. Bob, Cristina Căpușă, Irina-Draga Căruntu, Otilia Ciurea, Adrian Covic, Simona Giusca, Ina Kacso, Adelina Mihăescu, Andreea Niculescu, Bogdan Obrișcă, Dacian Tirinescu, Adalbert Schiller, Alexandra Vrabie, Yuriy Maslyennikov, Gener Ismail

**Affiliations:** 1 Department of Nephrology, “Grigore T. Popa” University of Medicine and Pharmacy, 700115 Iași, Romania; andreea.covic@gmail.com (A.C.); accovic@gmail.com (A.C.); 2 Clinical Hospital “Dr. C. I. Parhon”, 700503 Iași, Romania; irinadragacaruntu@gmail.com (I.-D.C.); simonaelizagiusca@gmail.com (S.G.); 3 Department of Nephrology, “Victor Babeș” University of Medicine and Pharmacy, 300041 Timișoara, Romania; bob.flaviu@umft.ro (F.R.B.); mihaescu.adelina@umft.ro (A.M.); timisoaraschiller@yahoo.com (A.S.); 4 Center for Molecular Research in Nephrology and Vascular Disease, Faculty of Medicine, “Victor Babeș” University of Medicine and Pharmacy, 300041 Timisoara, Romania; 5 Department of Nephrology, “Carol Davila” University of Medicine and Pharmacy, 050474 Bucharest, Romania; ccalexandr@yahoo.com (C.C.); otilia3091@yahoo.com (O.C.); 6 “Dr. Carol Davila” Clinical Hospital of Nephrology, 010731 Bucharest, Romania; calenic.andreea@gmail.com (A.N.); obriscabogdan@yahoo.com (B.O.); vornicu.alexandra@yahoo.com (A.V.); gener732000@yahoo.com (G.I.); 7 Department of Morpho-Functional Sciences I, “Grigore T. Popa” University of Medicine and Pharmacy, 700115 Iași, Romania; 8 Romanian Academy of Medical Sciences, 1 I.C. Bratianu Boulevard, 030171 Bucharest, Romania; 9 Academy of Romanian Scientists, 3, Ilfov Street, 050044 Bucharest, Romania; 10 Department of Nephrology, “Iuliu Hațieganu” University of Medicine and Pharmacy, 400012 Cluj-Napoca, Romania; inakacso@yahoo.com (I.K.); dacian_tirinescu@yahoo.com (D.T.); maslyennikov_yuriy@elearn.umf-cluj.ro (Y.M.); 11 Cluj County Emergency Hospital, 400006 Cluj-Napoca, Romania; 12 Fundeni Clinical Institute, 022328 Bucharest, Romania

**Keywords:** renal biopsy, glomerulonephritis, epidemiology, membranous nephropathy, focal segmental glomerulosclerosis, immunoglobulin A nephropathy, Romania, kidney disease registry, glomerular diseases

## Abstract

Glomerular diseases are a major cause of chronic kidney disease worldwide, yet epidemiological data from Eastern Europe, and Romania in particular, remain scarce. This study aimed to characterize the spectrum of biopsy-proven glomerulopathies in Romania through a multicenter national registry over a 10-year period. We retrospectively analyzed 4047 native kidney biopsies performed between 2014 and 2023 across four national nephrology reference centers. Patient demographics, clinical presentation, and histopathological diagnoses were collected and categorized into primary and secondary glomerular diseases, glomerulosclerosis, tubulointerstitial nephropathies, hereditary nephropathies, and vascular nephropathies. The mean patient age was 48 years, 54.8% were male, and 51.4% presented with nephrotic-range proteinuria. The most common primary glomerulopathies were membranous nephropathy (16.7%), immunoglobulin A nephropathy (15.6%), focal segmental glomerulosclerosis (8.8%), and membranoproliferative glomerulonephritis (10%). Among secondary glomerular diseases, lupus nephritis (9.3%), diabetic nephropathy (8.5%), and vasculitis (7.7%) were most frequent. Marked inter-center variability was observed, with a notably high prevalence of membranous nephropathy in Iași (31.1%). Over the study period, the incidence of focal segmental glomerulosclerosis increased while immunoglobulin A nephropathy declined. This study provides the first nationwide epidemiological assessment of biopsy-proven glomerular disease in Romania, revealing both similarities and distinctive differences compared to patterns reported in other European countries.

## 1. Introduction

Glomerular diseases are a major cause of chronic kidney disease (CKD) and end-stage renal disease (ESRD) worldwide, with significant implications for morbidity, mortality, and healthcare costs [1]. Despite advances in non-invasive biomarkers, renal biopsy remains the gold standard for diagnosing and classifying glomerular diseases, providing critical information for management and prognosis.

In Europe, national and regional biopsy registries have significantly contributed to understanding glomerular disease epidemiology. However, data from Eastern Europe remain limited, and Romania has historically been underrepresented in international nephrology registries. Previous Romanian studies have provided valuable insights, but were limited to single-center or regional cohorts, lacking national coverage and standardized protocols. For instance, Covic et al. (2006, 2021) reported a 10-year review of two, respectively one regional biopsy databases in northeast and western Romania [2,3], while Maslyennikov et al. (2025) presented data from a regional center in Cluj [4], and Pană et al. (2024) described trends from Romania’s largest reference center in Bucharest [5]. These efforts, though important, do not offer a unified national picture or assess regional variability using harmonized diagnostic criteria.

To address this gap, we established a multicenter Romanian Renal Biopsy Registry, aiming to systematically collect and analyze data from adult patients with biopsy-proven glomerulopathies across the country. This study presents the first comprehensive nationwide analysis, covering a 10-year period, to describe the demographic and histopathological landscape of glomerular disease in Romania.

## 2. Materials and Methods

### 2.1. Study Design and Setting

This retrospective, multicenter observational study analyzed all native kidney biopsies performed in adult patients (≥18 years) across four nephrology centers in Romania between January 2014 and December 2023. The four centers which participated represent the sole national reference centers for performing renal biopsies, covering a broad catchment population across Romania.

### 2.2. Inclusion and Exclusion Criteria

All native kidney biopsies performed for diagnostic purposes in patients aged 18 years or older were included. The main clinical indications for performing a kidney biopsy in adult patients included nephrotic-range proteinuria (>3.5 g/24 h), with or without preserved renal function; subnephrotic proteinuria (<3.5 g/24 h) in association with microscopic or macroscopic hematuria; and rapidly progressive glomerulonephritis (RPGN) or acute kidney injury (AKI) of unknown etiology, particularly when accompanied by active urinary sediment. Additional indications included persistent hematuria with dysmorphic red blood cells and/or red blood cell casts, systemic diseases with suspected renal involvement such as lupus erythematosus, ANCA-associated vasculitis, or amyloidosis, and the unexplained deterioration of renal function in the presence of urinary abnormalities. Biopsies were also indicated in cases of familial kidney disease or suspected hereditary nephropathies (e.g., Alport syndrome or Fabry disease), as well as in patients with plasma cell dyscrasias, monoclonal gammopathy of renal significance (MGRS), or suspected paraproteinemias. These indications were standardized and agreed upon by all participating centers during a consensus meeting organized by the Romanian Society of Nephrology, ensuring uniformity in biopsy practices across the national registry.

Biopsies of transplant kidneys, repeat biopsies from the same patient within the study period, and non-diagnostic biopsies (i.e., insufficient tissue for diagnosis) were excluded from analysis. Biopsies were considered adequate for diagnostic interpretation if the sample contained a minimum of eight glomeruli for light microscopy, with accompanying tissue for immunofluorescence and, when clinically indicated, electron microscopy. Based on these criteria, 299 biopsies were excluded from the final analysis due to insufficient tissue.

### 2.3. Biopsy Procedure and Histopathological Analysis

In all participating centers, kidney biopsies were performed using ultrasound-guided percutaneous techniques according to standardized protocols. Biopsy specimens were processed and evaluated by two independent, experienced pathologists in each center. Histopathological analysis included light microscopy, immunofluorescence staining (targeting IgG, IgA, IgM, C3, C1q, and, when indicated, kappa and lambda light chains), and electron microscopy where required. Diagnoses were established based on internationally accepted histopathological criteria.

### 2.4. Data Collection

For each patient, the following variables were systematically recorded at the time of biopsy: name, date of birth, sex, date of biopsy, age, serum creatinine, estimated glomerular filtration rate (eGFR), proteinuria, and the primary histopathological diagnosis. Only the principal diagnosis was included for analysis; secondary histopathological findings were not considered. Clinical data were extracted from standardized biopsy request forms and local electronic medical records.

### 2.5. Disease Classification

Histopathological diagnoses were categorized into six main groups: primary glomerular disease, secondary glomerular disease, glomerulosclerosis, tubulointerstitial nephropathy, hereditary nephropathy, and vascular nephropathy.

Classification followed internationally accepted definitions and diagnostic criteria as outlined in the KDIGO 2021 Clinical Practice Guideline for Glomerular Diseases [6] ensuring consistent diagnostic categorization across participating centers. Primary glomerular diseases included immunoglobulin A nephropathy (IgAN), membranous nephropathy (MN), focal segmental glomerulosclerosis (FSGS), global glomerulosclerosis, minimal change disease (MCD), membranoproliferative glomerulonephritis (MPGN), and C3 glomerulopathy (C3-GN). Secondary glomerular diseases comprised lupus nephritis, amyloidosis, diabetic nephropathy, vasculitis, and light-chain deposition disease.

### 2.6. Statistical Analysis

Descriptive statistics were used to summarize patient demographics and histopathological findings. Data are reported as means, medians, or percentages as appropriate. Incidence rates were expressed per million population per year (p.m.p./year). Biopsy incidence was calculated as the number of new native biopsies per million adult population per year (p.m.p./year), using population data from the 2021 Romanian census. Temporal trends and group differences were analyzed using the Cochran–Armitage test for trend or chi-square tests. A *p*-value < 0.05 was considered statistically significant. Statistical analyses were performed using IBM SPSS Statistics software, version X (IBM Corp., Armonk, NY, USA).

### 2.7. Ethical Considerations

The study was conducted in accordance with the Declaration of Helsinki and was approved by the Ethics Committees of all participating institutions. Patient confidentiality was maintained throughout the study, and data were anonymized prior to analysis.

## 3. Results

### 3.1. Overview of Biopsy Data and Patient Demographics

In Romania, native kidney biopsies are performed at four national reference centers: Bucharest (B)—comprising “Carol Davila” University Hospital and Fundeni Clinical Institute; Cluj-Napoca (CJ)—Cluj Emergency Hospital; Iași (IS)—“Dr. C.I. Parhon” Hospital; and Timișoara (TM)—Emergency Hospital Timișoara. Between 2006 and 2024, a total of 4346 biopsies were conducted across these centers. For the current analysis, we included 4047 biopsies performed during the 10-year period from 2014 to 2023, for which complete clinical and histopathological data were available.

The overall demographic and clinical profile of the patients is summarized in Table 1A. The mean age was 48.9 years, with a slight male predominance (54.8%). On average, patients had a serum creatinine level of 2.41 mg/dL, corresponding to an estimated glomerular filtration rate (eGFR) of 55.3 mL/min. Notably, 51.4% of patients presented with nephrotic-range proteinuria (>3 g/24 h), indicating a high burden of advanced glomerular disease at the time of biopsy. One-way ANOVA showed no difference in age, significant differences in the creatinine values from 2018 to 2019, proteinuria from 2014 to 2015, and the eGFR from 2014 to 2015 (Table 1B). Chi-square analysis showed significant differences between gender distribution across different time periods, with the 2020–2021 interval showing the lowest male renal biopsy percentage.

### 3.2. Distribution of Glomerular Diseases

The most common primary glomerular diseases diagnosed over the study period were membranous nephropathy (MN, 16.7%), IgA nephropathy (IgAN, 15.6%), focal segmental glomerulosclerosis (FSGS, 8.8%), minimal change disease (MCD, 7.6%), and MPGN (10.0%). There was substantial inter-center variability in disease distribution. For example, MN was particularly prevalent in Iași (31.1%), while FSGS incidence peaked in Timișoara (20.4%), and IgAN was most frequently diagnosed in Bucharest (19.3%) but was rare in Timișoara (2.1%) (Table 2). These differences may reflect regional referral patterns, urban-versus-rural population characteristics, and differing access to early nephrology care, as discussed later.

Among secondary glomerular diseases, lupus nephritis was the most common (9.3%), followed by diabetic nephropathy (8.5%) and vasculitis (7.7%). As with primary GN, their frequencies varied across centers—for example, diabetic nephropathy accounted for 10.9% of biopsies in Bucharest but only 1.6% in Iași, while vasculitis peaked at 10.9% in Timișoara. Other secondary and less frequent diagnoses are detailed in Table 2.

### 3.3. Incidence Trends over Time and by Region

To determine the incidence per million people and per year of specific glomerular diseases, the last census in Romania (2021) was used to assess the adult population from the surrounding counties of each Renal Biopsy Center.

Bucharest Center (B) was considered to serve a population of 6,798,507, Iasi Center (IS) a population of 3,075,428, Cluj Center (CJ) a population of 3,734,735, and Timisoara Center (TM) a population of 1,332,160.

**Figure 1:**
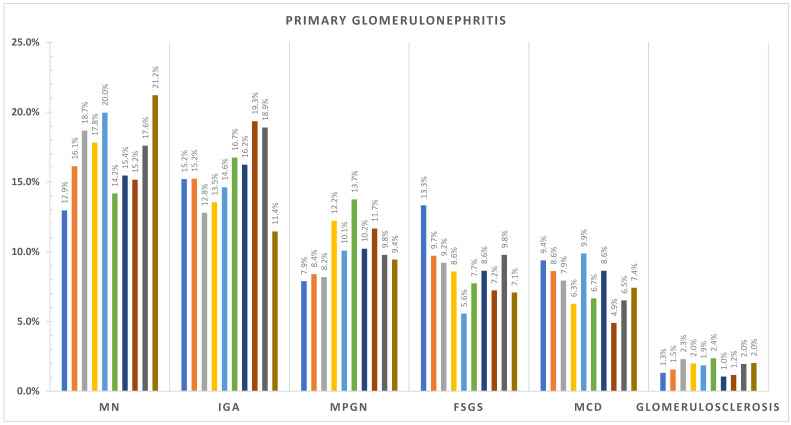
Primary glomerulonephritis incidence over the last 10 years (2023–2014 from left to right). IGA—IgA nephropathy; MN—membranous nephropathy; FSGS—focal segmental glomerulosclerosis; MCD—minimal change disease; MPGN—membranoproliferative glomerulonephritis.

The incidence per million population per year (p.m.p./year) for Romania is summarized in Table 3, while regional incidence data for each renal biopsy center—Bucharest, Iași, Cluj, and Timișoara—are presented in Table 4, Table 5, Table 6 and Table 7. At the national level, FSGS showed a clear upward trend, rising from 1.41 p.m.p./year in 2014 to 4.75 in 2023. In contrast, IgAN incidence initially increased from 2.28 in 2014 to a peak of 5.56 in 2016, followed by a gradual decline in the latter half of the decade, reaching 3.35 in 2021 and 2.74 in 2020. MN remained relatively stable, ranging from 3.61 to 6.49 p.m.p./year across the study period. These national-level trends are illustrated in Figure 1.

At the regional level, additional patterns emerged. In Timișoara, FSGS incidence rose sharply from 1.5 in 2014 to 6.76 in 2021 and 2023. Bucharest and Cluj exhibited similar increases. In contrast, IgAN was virtually absent in Iași and Timișoara after 2015, while Bucharest consistently reported the highest IgAN rates, reaching 10 p.m.p./year in 2023. Cluj showed lower but relatively stable IgAN incidence, with a slight rise in later years.

MN incidence showed limited variation over time but differed between centers: Iași reported the highest MN rates (e.g., 6.18 in 2022), while Cluj and Timișoara generally reported lower levels. MCD and MPGN demonstrated fluctuating patterns across centers without a consistent national trend.

**Figure 2:**
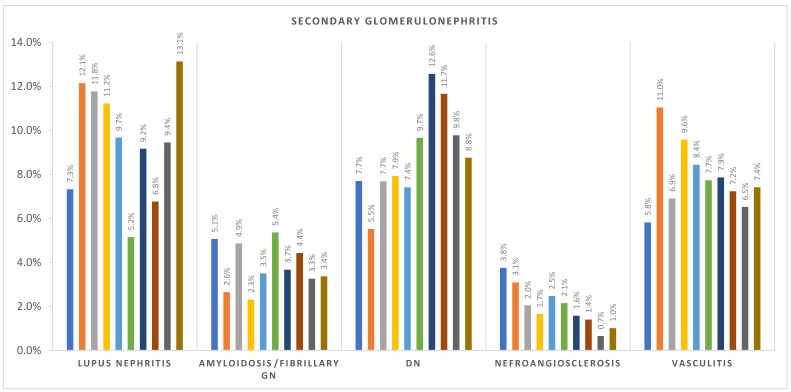
Secondary glomerulonephritis incidence over the last 10 years (2023–2014 from left to right). DN—diabetic nephropathy.

**Figure 3:**
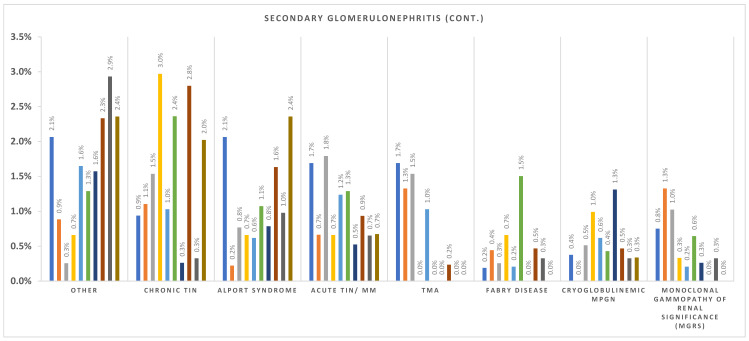
Secondary glomerulonephritis incidence over the last 10 years (2023–2014 from left to right). TIN—tubulointerstitial nephritis, TMA—thrombotic microangiopathy.

Among secondary glomerular diseases, vasculitis incidence increased modestly over time. In Timișoara, it rose from 0.75 in 2014 to 4.5 in 2022, with similar upward trends observed in other centers. Lupus nephritis and diabetic nephropathy incidence remained stable overall but varied in frequency by region—trends depicted in Figure 2 and Figure 3, summarizing the evolving landscape of secondary glomerular diseases in Romania.

## 4. Discussion

We had hypothesized that the Romanian renal biopsy registry would reveal a distinct epidemiological pattern of biopsy-proven glomerulopathies, shaped by regional genetic, environmental, and healthcare factors. Our findings confirm this hypothesis, showing that membranous nephropathy (MN), IgA nephropathy (IgAN), and focal segmental glomerulosclerosis (FSGS) are the predominant primary glomerulopathies, while lupus nephritis (LN), diabetic nephropathy (DN), and vasculitis are the most common secondary glomerular diseases. Compared to other national registries, our data suggest notably high MN prevalence, stable MPGN incidence, and regional variations in vasculitis and DN prevalence.

### 4.1. Comparison with Global Data

According to our data, IgAN accounts for 15.6% of biopsy-proven GN cases in Romania, which is comparable to Europe (ranging from 3.6% in Belgium [7] to 34.9% in Finland [8]) and the reported prevalence in Spain [9] (14.6%), Poland [10] (20.0%), and Germany [11] (20.0%). IgAN in our study is more prevalent than in other Eastern European registries (Serbia [12]—7.7%). Also, IgAN was more prevalent than in America (7.7% in a study performed in Cleveland Clinic centers [13] and 10.3% in a large study conducted in North Carolina [14]). However, it remains significantly lower than in Asian registries, where IgAN prevalence reaches 35.8% in China [15] and 31.0% in Japan [16], reflecting genetic and environmental predispositions.

Despite the use of standardized biopsy indications across all participating centers, notable inter-center differences were observed in the incidence of specific glomerular diseases—most prominently for IgA nephropathy, which ranged from 19.3% in Bucharest to as low as 2.1% in Timișoara. These differences likely reflect factors beyond biopsy policy, such as variation in referral patterns, access to nephrology care, and population structure. For example, Bucharest serves a predominantly urban population with greater proximity to tertiary care and earlier nephrology referral, which may lead to the increased detection of milder forms of glomerular disease such as IgA nephropathy. In contrast, centers serving more rural or dispersed populations may receive patients at more advanced disease stages or with syndromes more likely to prompt urgent biopsy, potentially underrepresenting indolent or subclinical presentations like IgA nephropathy. Similar geographic and socioeconomic disparities have been reported in other national registries, such as in Spain [9] and China [15], where earlier referral and access to nephrology services have been associated with higher detection rates of IgA nephropathy. Thus, the observed variability in disease incidence likely reflects differences in health system access and referral behavior, rather than divergent clinical decision-making or diagnostic thresholds. 

MN in Romania (16.7%) is among the highest in Europe (ranging between 4.7% in Lithuania [17] and 14.4% in Italy [18]), significantly exceeding the rates observed in Germany [11] (9.0%), Spain [9] (9.9%), and Poland [10] (11.2%), but similar to Italy [18] (14.4%) and Serbia [12] (12.6%). Interestingly, our study found the highest MN prevalence in Iasi (31.1%), further suggesting possible regional environmental or genetic factors influencing disease distribution. In America, high MN prevalence was reported in some studies (20.3% in Arizona [19] and 12.9% in North Carolina [14]), while other major studies showed a significantly lower prevalence (5.1% in Cleveland Clinic centers [13] and 5.3% in Minnesota [20]). In Asia, high MN prevalence has been reported in China [21] (24.9%), Nepal [22] (14.7%), and Pakistan [23] (20%).

FSGS rates in Romania (8.8%) are similar to those reported in Europe (7.8% in Lithuania [17], 9% in Belgium [7], 8% in Spain [9]) but greatly lower than in North America (22.3% in Arizona [15] and 25.3% in North Carolina [14]) and South America (25% in Brazil [24], 22% in Columbia [25]), where FSGS has emerged as the predominant glomerulopathy. This discrepancy may be due to differences in biopsy practices, genetic predisposition, and environmental influences such as obesity and metabolic syndrome, which are key risk factors for FSGS.

The reported prevalence of MPGN in Romania was 10%; higher than that reported in Europe (3.2% in the Czech Republic [26], 4.6% in Poland [10], 6.8% in Serbia [12], and 3.9% in Spain [9]). The highest MPGN prevalence in Europe outside Romania has been reported in Lithuania [17] (7.4%) and Italy [18] (7%). Given that, in our registries, MPGN in the context of cryoglobulinemia was recorded distinctly, we hypothesize that among these cases of immune complex-mediated MPGN a significant proportion can be attributed to an underlying alternative complement pathway dysregulation. Nonetheless, given that this data is recorded in a biopsy registry, the availability of complement system activity evaluation could not be assessed and definitive proof for this hypothesis is lacking.

Regarding secondary glomerular diseases, lupus nephritis (9.3%) rates in Romania are comparable to those in Europe (the lowest is in Lithuania [17]—2.3%, the highest in a smaller study in Spain [27]—22.4%, with 9.96% in Serbia [12], 8.4% in Poland [10], and 7.1% in the Czech Republic [26]), but lower than in South America (22% in Brazil [24] and 17% in Columbia [25]). Diabetic nephropathy (8.5%) is more frequent than in other European countries (3.7% in Poland [10], 4.1% in the Czech Republic [26], and 4.8% in Spain [9]), but significantly lower than in North America (14.59% in the Cleveland Clinic study [13]). The prevalence of vasculitis in Romania (7.7%) was similar to that of Europe (6.8% in Spain [9], 8.37% in the Netherlands [28], and 5.7% in the Czech Republic [26]) and North America (7.9% in Arizona [19] and 7.9% in North Carolina [14]) but higher than in Asia (1% in China [15] and 4.8% in Japan [16]).

### 4.2. Temporal Trends and Potential Explanations

The temporal analysis of demographic and clinical parameters revealed relative stability in age distribution over the past decade, while significant variations in serum creatinine, proteinuria, eGFR, and gender distribution across specific intervals suggest evolving patterns in disease severity at presentation and possibly shifting thresholds or referral behaviors influencing biopsy practices.

A notable trend in our study is the increase in FSGS incidence over the last decade, paralleling global trends in developed countries. This rise could be attributed to improved recognition and biopsy practices, an increased prevalence of risk factors such as obesity and hypertension, or a genuine shift in glomerular disease epidemiology. Conversely, IgAN shows a decreasing trend, which may reflect evolving biopsy indications, better early management, or changes in environmental exposure affecting disease onset.

The incidence of vasculitis and membranoproliferative glomerulonephritis (MPGN) has remained stable or has slightly increased, mirroring trends in other European registries. The persistence of vasculitis cases suggests an aging population with increasing detection of ANCA-associated glomerulonephritis. Meanwhile, the stable MPGN rates contrast with the decreasing trend reported in North America, where improved infection control and reduced hepatitis C prevalence have led to a decline in MPGN cases.

### 4.3. Factors Influencing Findings

Several factors could contribute to the observed differences in disease prevalence and trends in Romania compared to other countries. Biopsy practices vary across different healthcare systems and, in some European countries, biopsy rates are higher in elderly patients and those with advanced chronic kidney disease, which may lead to different glomerular disease distributions. Environmental and genetic factors likely play a role as well. The high prevalence of IgAN in Romania is consistent with other European populations, suggesting common genetic predispositions. Additionally, healthcare access and referral patterns may influence biopsy rates for conditions such as diabetic nephropathy, which is often diagnosed clinically without biopsy in some healthcare settings.

Regarding the observed variability in disease frequencies across Romanian centers, several factors may contribute. Certain centers may have a lower threshold for performing kidney biopsies, particularly in patients presenting with nephrotic-range proteinuria or atypical clinical syndromes, leading to higher detection rates of specific glomerular diseases. For example, the notably high prevalence of membranous nephropathy in Iași (31.1%) may reflect a center-specific practice of routinely biopsying all patients with nephrotic syndrome, whereas in other centers, elderly patients with presumed diabetic nephropathy may not undergo biopsy. Similarly, the lower detection of IgA nephropathy in Timișoara may be due to less frequent biopsies in young adults presenting with isolated hematuria or mild proteinuria. Additionally, inter-observer variability in histopathological interpretation may contribute to differences in reported frequencies. In the absence of formal centralized pathology review or national nephropathology specialization, diagnoses may be inconsistently classified across centers.

### 4.4. Study Limitations

While this study provides valuable epidemiological insights, several limitations should be acknowledged. The biopsy cohort represents only patients who underwent kidney biopsy, potentially underestimating diseases with less severe clinical presentations. Regional differences in biopsy indications may influence disease distribution, as some centers may perform biopsies more frequently than others. Furthermore, follow-up data on disease progression and treatment response were not included, limiting conclusions on disease outcomes.

### 4.5. Clinical Implications and Future Directions

These findings have important implications for clinical practice and research. The increasing incidence of FSGS warrants further investigation into potential environmental and genetic risk factors. The stable prevalence of MPGN and vasculitis suggests the need for continued surveillance and early detection strategies. Additionally, the decreasing trend of IgAN highlights the potential role of improved early disease recognition and management. Future studies should aim to expand the biopsy registry, incorporate long-term follow-up data, and explore genetic and environmental determinants of glomerular disease patterns in Romania. Establishing a collaborative Eastern European biopsy registry could further enhance our understanding of regional trends and contribute to global nephrology research.

## 5. Conclusions

This study provides the most comprehensive biopsy-based epidemiological analysis of glomerular diseases in Romania to date. The results demonstrate both consistencies and differences with European and global patterns, reflecting the interplay of genetic, environmental, and healthcare factors. The observed trends, particularly the increasing incidence of FSGS and the declining prevalence of IgAN, emphasize the need for continuous surveillance and further research to optimize diagnosis, treatment, and prevention strategies in glomerular disease management.

## Figures and Tables

**Table 1:** A. Demographic and clinical features of patients who underwent native kidney biopsy in Romania. Values include mean age, sex distribution, serum creatinine, estimated glomerular filtration rate (eGFR), and proteinuria status. Data are presented as overall totals and stratified by renal biopsy center. B—Bucharest Center, CJ—Cluj Center, IS—Iasi Center, TM—Timisoara Center. B. Demographic data across different time periods.

**A**					
	**All Patients**	**B**	**CJ**	**IS**	**TM**
	N = 4346	N = 3004	N = 556	N = 502	N = 284
Age (years)	48.88 ± 15.46	49.86 ± 15.56	47.15 ± 15.64	43.25 ± 13.79	51.82 ± 14.26
Creatinine (mg/dL)	2.41 ± 2.46	2.33 ± 2.2	2.22 ± 2.25	2.99 ± 3.5	2.59 ± 3.01
Proteinuria (g/24 h)	4.98 ± 5.62	4.61 ± 5.35	6.47 ± 6.36	5.22 ± 5.53	5.41 ± 6.34
eGFR (mL/min)	55.31 ± 37.34	53.43 ± 35.37	63.74 ± 40.69	57.63 ± 43.15	54.98 ± 37.36
>3 g/day (%)	51.4%	48.9%	62.2%	55.0%	49.3%
Male (%)	54.8%	53.6%	52.7%	61.4%	60.2%
**B**					
	**2022–2023**	**2020–2021**	**2018–2019**	**2016–2017**	**2014–2015**
Age	50 ± 15	49 ± 15	48 ± 15	49 ± 14	47 ± 15
Creatinine (mg/dL)	2.3 ± 2.4	2.4 ± 2.5	2.2 ± 2.3 ^a^	2.6 ± 2.6	2.5 ± 2.4
Proteinuria	5.1 ± 5.7	5.5 ± 5.9	5.2 ± 5.6	4.7 ± 6.3	4.3 ± 4.2 ^b^
eGFR (mL/min)	56 ± 37	54 ± 38	59 ± 38	51 ± 35 ^c^	51 ± 34 ^d^
Gender (%M)	52.8%	48.8%	54.5%	59.4%	58.1%

Data presented as mean ± standard deviation. ^a^ Significantly different to 2016–2017. ^b^ Significantly different to 2018–2019 and 2020–2021. ^c^ Significantly different to 2022–2023 and 2018–2019. ^d^ Significantly different to 2018–2019.

**Table 2:** The cumulative frequency of primary and secondary glomerular diseases, glomerulosclerosis, and other histopathological categories across the four Romanian renal biopsy centers (Bucharest, Cluj, Iași, and Timișoara) from 2014 to 2023. The values are presented as percentages of total biopsies at each center. Red—highest for each diagnosis between 2014 and 2023; blue—primary GN. IgA—IgA nephropathy; MN—membranous nephropathy; FSGS—focal segmental glomerulosclerosis; MCD—minimal change disease; MPGN—membranoproliferative glomerulonephritis; DN—diabetic nephropathy; TMA—thrombotic microangiopathy; TIN—tubulointerstitial nephritis; MM—multiple myeloma.

	B	CJ	IS	TM	Total
**IgA**	19.3%	13.7%	3.4%	2.1%	15.6%
**MN**	15.5%	14.0%	31.1%	8.8%	16.7%
**FSGS**	8.1%	11.3%	3.4%	20.4%	8.8%
**MCD**	7.9%	11.9%	0.0%	9.9%	7.6%
**C3/DDD**	0.2%	0.0%	0.0%	0.4%	0.2%
**Cryoglobulinemic MPGN**	0.7%	0.0%	0.6%	0.7%	0.6%
**IC-MPGN**	7.2%	7.2%	27.5%	14.1%	10.0%
**Lupus nephritis**	8.9%	11.3%	9.4%	9.9%	9.3%
**Amyloidosis/Fibrillary GN**	5.0%	0.17%	2.6%	3.5%	4.0%
**Alport syndrome**	1.3%	1.4%	0.0%	0.0%	1.1%
**Fabry disease**	0.5%	0.0%	0.0%	0.4%	0.4%
**DN**	10.9%	3.4%	1.6%	5.3%	8.5%
**Nephroangiosclerosis**	2.7%	2.0%	0.6%	0.0%	2.2%
**TMA**	0.8%	0.0%	0.0%	2.5%	0.7%
**Vasculitis**	6.8%	9.2%	9.4%	10.9%	7.7%
**Monoclonal gammopathy of renal significance (MGRS)**	0.3%	0.4%	0.0%	3.9%	0.5%
**Acute TIN/MM**	0.4%	4.53%	0.0%	2.8%	1.1%
**Chronic TIN**	1.1%	3.8%	1.2%	1.4%	1.5%
**Other**	1.2%	5.2%	1.2%	0.4%	1.7%
**Glomerulosclerosis**	1.1%	0.5%	8.2%	2.8%	2.0%
	100.0%	100.0%	100.0%	100.0%	100.0%

**Table 3:** Annual incidence per million adult population of major glomerular diseases across all four centers combined. Blue—primary GN. IgA—IgA nephropathy; MN—membranous nephropathy; FSGS—focal segmental glomerulosclerosis; MCD—minimal change disease; MPGN—membranoproliferative glomerulonephritis; DN—diabetic nephropathy; TMA—thrombotic microangiopathy; TIN—tubulointerstitial nephritis; MM—multiple myeloma; tubulointerstitial nephritis; MM—multiple myeloma.

	2023	2022	2021	2020	2019	2018	2017	2016	2015	2014
IgA	5.42	4.62	3.35	2.74	4.75	5.22	4.15	5.56	3.88	2.28
MN	4.62	4.89	4.89	3.61	6.49	4.42	3.95	4.35	3.61	4.22
FSGS	4.75	2.94	2.41	1.74	1.81	2.41	2.21	2.07	2.01	1.41
MCD	3.35	2.61	2.07	1.27	3.21	2.07	2.21	1.41	1.34	1.47
C3/DDD	0.2	0	0	0	0	0	0.07	0.07	0	0
Cryoglobulinemic MPGN	0.13	0	0.13	0.2	0.2	0.13	0.33	0.13	0.07	0.07
MPGN	2.81	2.54	2.14	2.48	3.28	4.28	2.61	3.35	2.01	1.87
Lupus nephritis	2.61	3.68	3.08	2.28	3.15	1.61	2.34	1.94	1.94	2.61
Amyloidosis/Fibrillary GN	1.81	0.8	1.27	0.47	1.14	1.67	0.94	1.27	0.67	0.67
Alport syndrome	0.74	0.07	0.2	0.13	0.2	0.33	0.2	0.47	0.2	0.47
Fabry disease	0.07	0.13	0.07	0.13	0.07	0.47	0	0.13	0.07	0
DN	2.74	1.67	2.01	1.61	2.41	3.01	3.21	3.35	2.01	1.74
Nefroangiosclerosis	1.34	0.94	0.54	0.33	0.8	0.67	0.4	0.4	0.13	0.2
TMA	0.6	0.4	0.4	0	0.33	0	0	0.07	0	0
Vasculitis	2.07	3.35	1.81	1.94	2.74	2.41	2.01	2.07	1.34	1.47
Monoclonal gammopathy of renal significance (MGRS)	0.27	0.4	0.27	0.07	0.07	0.2	0.07	0	0.07	0
Acute TIN/MM	0.6	0.2	0.47	0.13	0.4	0.4	0.13	0.27	0.13	0.13
Chronic TIN	0.33	0.33	0.4	0.6	0.33	0.74	0.07	0.8	0.07	0.4
Other	0.74	0.27	0.07	0.13	0.54	0.4	0.4	0.67	0.6	0.47
Glomerulosclerosis	0.47	0.47	0.6	0.4	0.6	0.74	0.27	0.33	0.4	0.4

**Table 4:** Bucharest Center (B). Incidence per million people per year; blue—primary GN. IgA—IgA nephropathy; MN—membranous nephropathy; FSGS—focal segmental glomerulosclerosis; MCD—minimal change disease; MPGN—membranoproliferative glomerulonephritis; DN—diabetic nephropathy; TMA—thrombotic microangiopathy; TIN—tubulointerstitial nephritis; MM—multiple myeloma.

	2023	2022	2021	2020	2019	2018	2017	2016	2015	2014
IgA	10	8.53	6.32	4.56	9.12	9.86	8.24	10.88	7.65	4.27
MN	7.65	7.35	7.8	5.74	10.74	6.62	5.44	6.03	3.82	5.3
FSGS	6.32	4.12	3.38	3.09	2.35	4.12	3.68	2.5	3.24	0.88
MCD	5.59	4.27	2.94	1.62	4.85	3.09	3.68	2.35	2.5	2.06
C3/DDD	0.44	0	0	0	0	0	0.15	0.15	0	0
Cryoglobulinemic MPGN	0.15	0	0.29	0.44	0.29	0.29	0.74	0.15	0.15	0.15
MPGN	5.3	3.38	2.65	3.38	4.41	4.27	1.91	2.5	1.62	1.77
Lupus nephritis	4.12	5.44	5.3	3.97	4.56	2.94	3.24	2.94	3.53	1.91
Amyloidosis/Fibrillary GN	3.82	1.47	2.5	0.74	2.06	3.24	1.77	2.5	1.32	1.32
Alport syndrome	1.18	0.15	0.15	0.29	0.29	0.59	0.44	1.03	0.29	1.03
Fabry disease	0.15	0.15	0.15	0.29	0.15	1.03	0	0.29	0.15	0
DN	4.12	2.65	3.82	3.24	4.85	6.32	6.91	7.35	4.41	3.53
Nephroangiosclerosis	2.06	1.91	1.18	0.74	1.77	1.03	0.74	0.59	0.29	0.29
TMA	1.03	0.59	0.74	0	0.59	0	0	0.15	0	0
Vasculitis	3.38	4.71	2.5	2.5	4.27	3.24	3.09	2.94	1.47	1.62
Monoclonal gammopathy of renal significance (MGRS)	0	0.74	0.44	0.15	0	0	0	0	0.15	0
Acute TIN/MM	0.15	0.15	0.44	0.15	0.44	0.15	0	0.29	0	0
Chronic TIN	0.15	0.29	0.59	1.18	0.59	1.03	0	0.44	0	0.29
Other	0.74	0.44	0.15	0	0.74	0.44	0.74	0.29	0.74	0.29
Glomerulosclerosis	0.74	0.88	0.59	0.29	0.59	0.88	0.15	0.29	0.15	0.29

**Table 5:** Iasi Center (B). Incidence per million people per year; blue—primary GN. IgA—IgA nephropathy; MN—membranous nephropathy; FSGS—focal segmental glomerulosclerosis; MCD—minimal change disease; MPGN—membranoproliferative glomerulonephritis; DN—diabetic nephropathy; TMA—thrombotic microangiopathy; TIN—tubulointerstitial nephritis; MM—multiple myeloma.

	2022	2021	2020	2019	2018	2017	2016	2015	2014	2013
IgA	0	0	0	0	0	0.33	0.33	0.98	1.3	1.3
MN	6.18	4.55	2.93	4.88	3.58	3.58	4.23	6.18	3.58	4.88
FSGS	0.98	0.33	0	0.33	0	0	0.33	0.33	0.98	0.98
Cryoglobulinemic MPGN	0	0	0	0	0	0	0.33	0	0	0
MPGN	1.95	2.28	2.28	3.9	7.48	7.15	5.2	4.88	3.25	2.28
Lupus nephritis	0	0.33	1.95	1.63	0.33	0.98	0.98	0	3.9	2.6
Amyloidosis/Fibrillary GN	0	0	0.33	0.98	0.65	0.33	0.65	0	0.33	0.65
DN	0.65	0.33	0.33	0	0	0	0	0	0	0.33
Nephroangiosclerosis	0	0	0	0	0	0	0.65	0	0.33	0
Vasculitis	2.28	1.63	0.65	1.63	0	0.98	1.63	1.3	1.63	0.33
Chronic TIN	0	0.33	0	0	0	0	0.98	0	0.33	0.33
Other	0	0	0	0	0	0	0.65	0	0.33	0.33
Glomerulosclerosis	0.33	1.3	0.65	1.3	0.98	0.98	0.98	1.63	0.65	0.98

**Table 6:** Cluj Center (CJ). Incidence per million people per year; red—lowest, dark green—highest for each diagnosis between 2014 and 2023; blue—primary GN. IgA—IgA nephropathy; MN—membranous nephropathy; FSGS—focal segmental glomerulosclerosis; MCD—minimal change disease; MPGN—membranoproliferative glomerulonephritis; DN—diabetic nephropathy; TMA—thrombotic microangiopathy; TIN—tubulointerstitial nephritis; MM—multiple myeloma.

	2023	2022	2021	2020	2019	2018	2017	2016	2015	2014
IgA	2.95	2.95	1.87	2.68	2.41	2.95	1.34	2.14	0.8	0.27
MN	2.95	0.54	1.34	1.61	2.14	1.87	2.68	2.41	2.14	3.21
FSGS	5.09	0.8	0.8	1.07	1.61	0.27	1.34	2.14	1.07	2.68
MCD	2.68	1.61	2.14	1.07	3.75	1.61	1.07	1.34	0.8	1.61
MPGN	0.8	1.61	0.8	1.34	0.54	1.61	0	2.14	0.8	1.07
Lupus nephritis	1.61	4.28	1.07	0	1.87	0.54	1.61	1.61	1.07	3.21
Alport syndrome	0.8	0	0.54	0	0.27	0.27	0	0	0.27	0
DN	2.41	0.8	0.27	0.27	0.27	0.27	0.27	0	0	0.54
Nephroangiosclerosis	1.61	0.27	0	0	0	0.8	0.27	0	0	0
Vasculitis	1.61	1.34	0.8	1.61	1.34	1.87	1.34	1.07	1.34	1.34
Monoclonal gammopathy of renal significance (MGRS)	0.27	0	0	0	0	0.27	0	0	0	0
Acute TIN/MM	1.61	0.27	1.07	0.27	0.54	1.34	0.27	0.54	0.54	0.54
Chronic TIN	1.07	0.27	0.27	0.27	0.27	1.07	0.27	1.07	0.27	0.8
Other	1.34	0.27	0	0.54	0.8	0.8	0.27	1.61	1.07	1.07
Glomerulosclerosis	0.27	0	0.27	0	0.27	0	0	0	0	0

**Table 7:** Timisoara Center (TM). Incidence per million people per year; red—lowest, dark green—highest for each diagnosis between 2014 and 2023; blue—primary GN. IgA—IgA nephropathy; MN—membranous nephropathy; FSGS—focal segmental glomerulosclerosis; MCD—minimal change disease; MPGN—membranoproliferative glomerulonephritis; DN—diabetic nephropathy; TMA—thrombotic microangiopathy; TIN—tubulointerstitial nephritis; MM—multiple myeloma.

	2023	2022	2021	2020	2019	2018	2017	2016	2015	2014
IgA	1.5	0	0	0	0	0	0	0	0	0
MN	4.5	1.5	0.75	0	0.75	2.25	0.75	1.5	0.75	3
FSGS	6.76	7.51	6.76	0.75	3	5.25	2.25	3.75	2.25	1.5
MCD	1.5	3	2.25	3	0.75	3	3	0	0	1.5
C3/DDD	0	0	0	0	0	0	0	0	0	0
Cryoglobulinemic MPGN	0.75	0	0	0	0.75	0	0	0	0	0
MPGN	2.25	2.25	3	1.5	3.75	4.5	3	6.76	0.75	1.5
Lupus nephritis	3.75	1.5	3.75	0.75	3	0.75	3	0	0.75	1.5
Amyloidosis/Fibrillary GN	0.75	1.5	1.5	0.75	0	0.75	0.75	0	0.75	0
Fabry disease	0	0.75	0	0	0	0	0	0	0	0
DN	3	1.5	1.5	0	1.5	0.75	0	0	0	0
TMA	1.5	1.5	0.75	0	0.75	0	0	0	0	0
Vasculitis	1.5	4.5	1.5	3	1.5	5.25	0.75	1.5	0.75	0.75
Monoclonal gammopathy of renal significance (MGRS)	2.25	0.75	0.75	0	0.75	1.5	0.75	0	0	0
Acute TIN/MM	1.5	0.75	0	0	0.75	0	0.75	0	0	0
Chronic TIN	0	1.5	0	0	0	0	0	1.5	0	0
Other	0.75	0	0	0	0	0	0	0	0	0
Glomerulosclerosis	0.75	0	0	1.5	0	1.5	0	0	0	1.5

## Data Availability

The data presented in this study are not publicly available due to privacy restrictions. This is in accordance with the *MDPI Data Availability Policy*. Upon reasonable request, the corresponding author can provide further information about the data, subject to applicable restrictions.

## Abbreviations

The following abbreviations are used in this manuscript:

CKD	Chronic Kidney Disease
ESRD	End-Stage Renal Disease
GN	Glomerulonephritis
IgAN	Immunoglobulin A Nephropathy
MN	Membranous Nephropathy
FSGS	Focal Segmental Glomerulosclerosis
MCD	Minimal Change Disease
MPGN	Membranoproliferative Glomerulonephritis
C3-GN	C3 Glomerulopathy
LN	Lupus Nephritis
DN	Diabetic Nephropathy
RPGN	Rapidly Progressive Glomerulonephritis
AAV	ANCA-Associated Vasculitis
EM	Electron Microscopy
IF	Immunofluorescence
eGFR	Estimated Glomerular Filtration Rate
p.m.p./year	Per Million Population Per Year
